# Supplementary material for: Inferring histology-associated gene expression gradients in spatial transcriptomic studies
Source: Nat Commun. 2024 Aug 23;15:7280. doi: 10.1038/s41467-024-50904-x (PMC11343836; doi:10.1038/s41467-024-50904-x)
Supplement: Supplementary file 1 — Supplementary Information [file 41467_2024_50904_MOESM1_ESM.pdf]

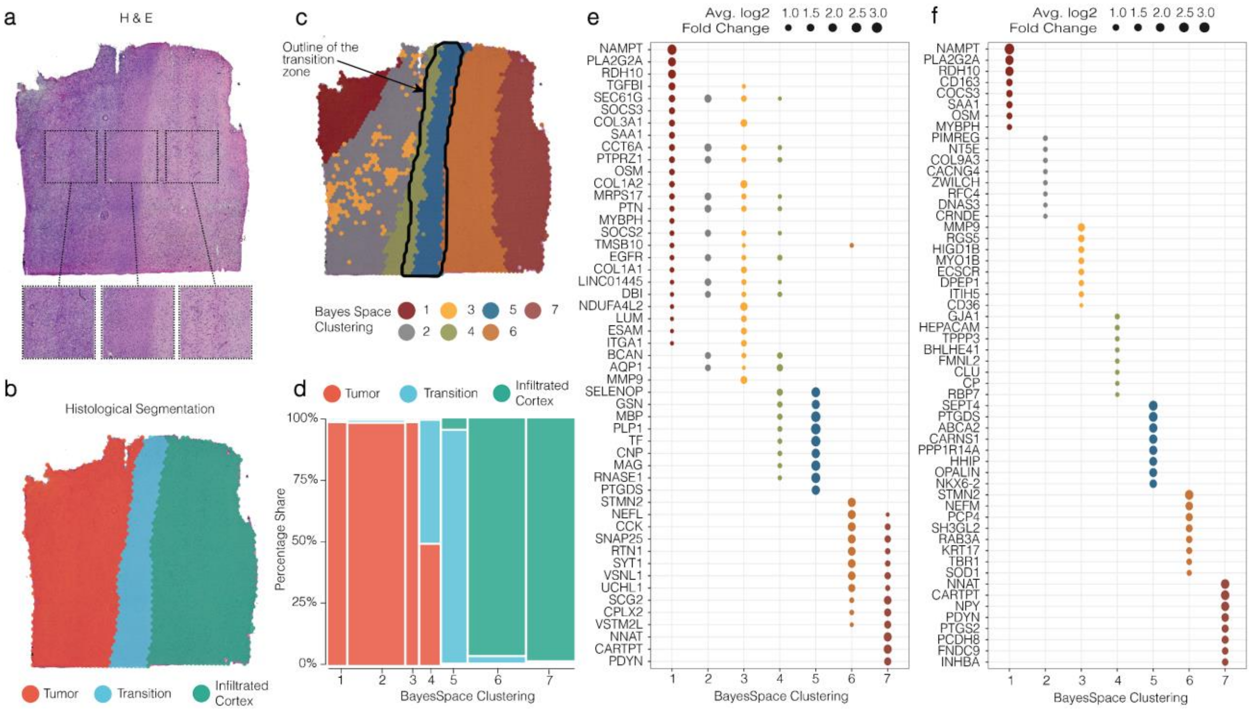

Supplementary Figure 1: a) H&E Image of the sample UKFT269. Areas that guided our manual histological classification are highlighted. b) Surface plot illustrates the histological incorporation in form of grouping data variables. The barcode-spots are colored according to their histological group. c) Surface plot that shows the suggested clustering of the BayesSpace algorithm. Cluster 4 and particularly cluster 5 overlap with the transition zone. The spatial segregation of both clusters (in contrast to e.g. cluster 3) suggests border like characteristics. d) A mosaic-plot illustrates the large overlap of spots between the transition zone and cluster 5 as well as the partial overlap between transition zone and cluster 4. e) Dotplot of the 8 most significant genes prior to filtering for cluster unique marker genes. The large number of shared marker genes renders DEA a suboptimal standalone-tool for spatial gene expression analysis. f) Dotplot of the 8 most significant genes after filtering for unique marker-genes. See Supplementary Figure 2 for spatial expression and gradients of these genes.

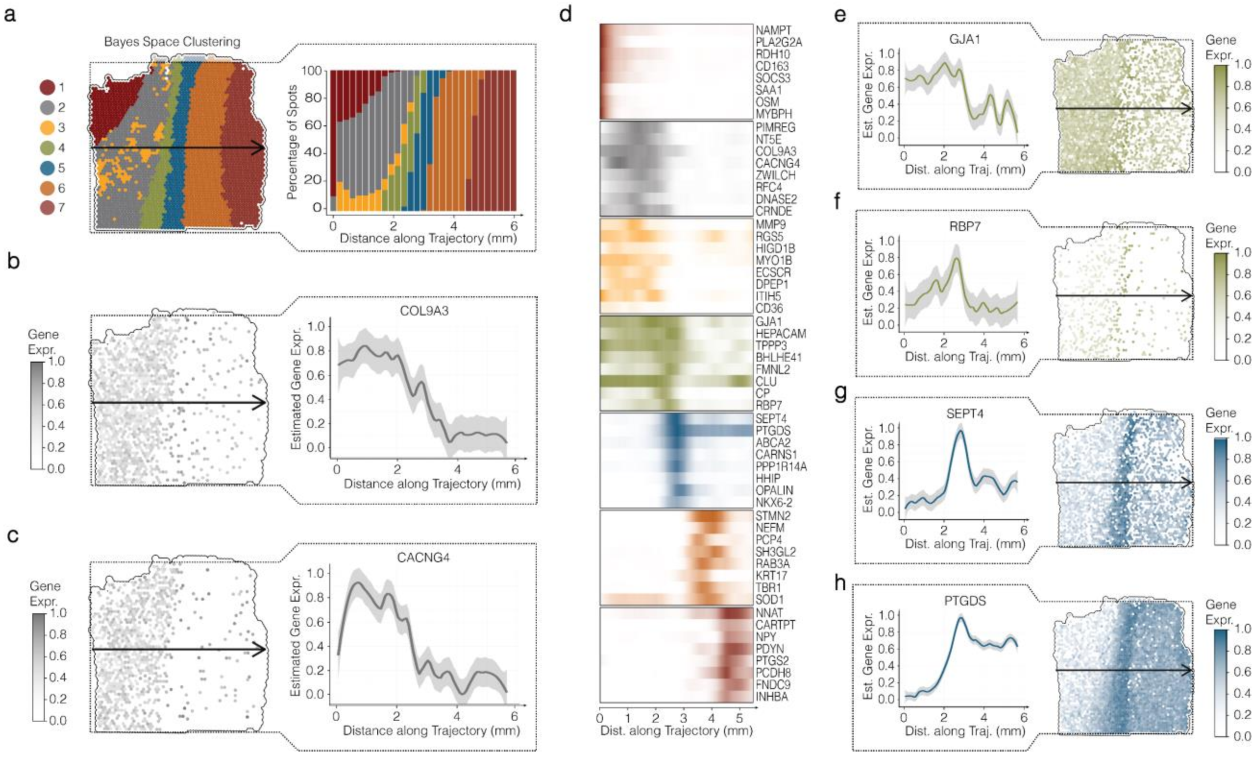

Supplementary Figure 2: Visualization of Gene Expression Gradients for Unique BayesSpace Marker Genes Identified through Differential Expression Analysis (DEA). a) A surface plot provides a comprehensive representation of the trajectory's course and extent, while a corresponding barplot illustrates the dynamic fluctuations in the proportion of the BayesSpace cluster along this trajectory. b-c) Two surface plots elucidate the expression patterns of specific genes, closely aligned with the spatial boundaries of the cluster for which they were identified as marker genes. d) Seven heatmaps depict the gradients of the eight most statistically significant group-specific marker genes, as identified by DEA for the clustering recommended by BayesSpace (see Supplementary Figure 1f). These heatmaps reveal distinctive expression patterns, with some genes exhibiting elevated expression precisely where the cluster proportion is high, while others demonstrate unique, non-conforming patterns. e-h) These examples underscore the diversity in gene expression patterns, even among genes identified as unique marker genes, emphasizing the complexity and multifaceted nature of gene expression.

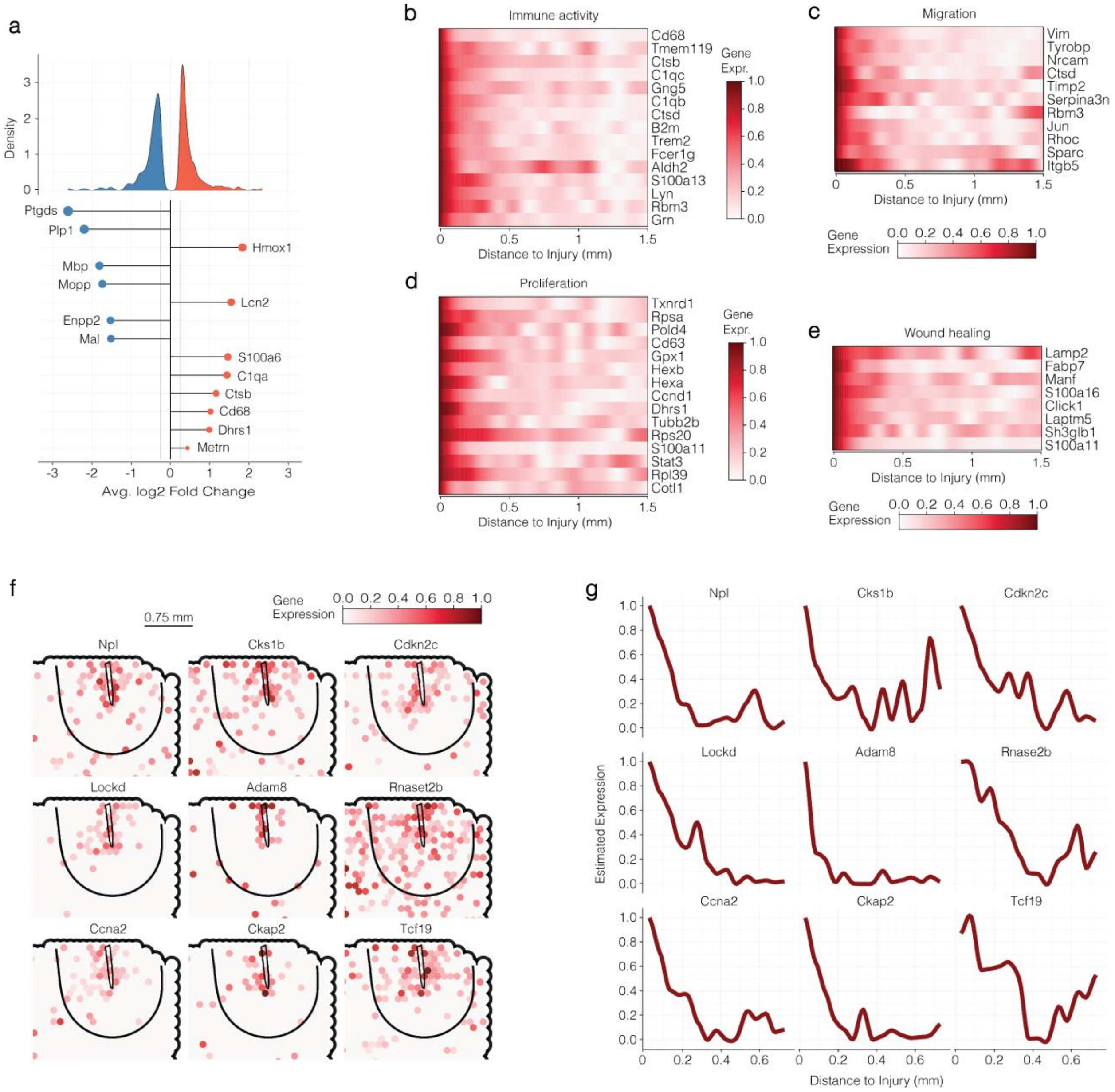

Supplementary Figure 3: Spatially Resolved Expression Gradients of Key Pathways and Cell Types Associated with Stab Wounds. a) DEA results for marker genes displayed in the main figure. b-e) Gradient heatmaps depict the gene expression patterns of multiple genes identified by spatial annotation screening as exhibiting non-random, descending expression profiles as a function of distance, up to 1.5mm from the stab wounds. f-g) Close-up illustrations of select genes not detected by DEA but displaying significant gradients when referenced to the annotated injuries. These findings suggest their involvement in orchestrating a response to the stab wounds.

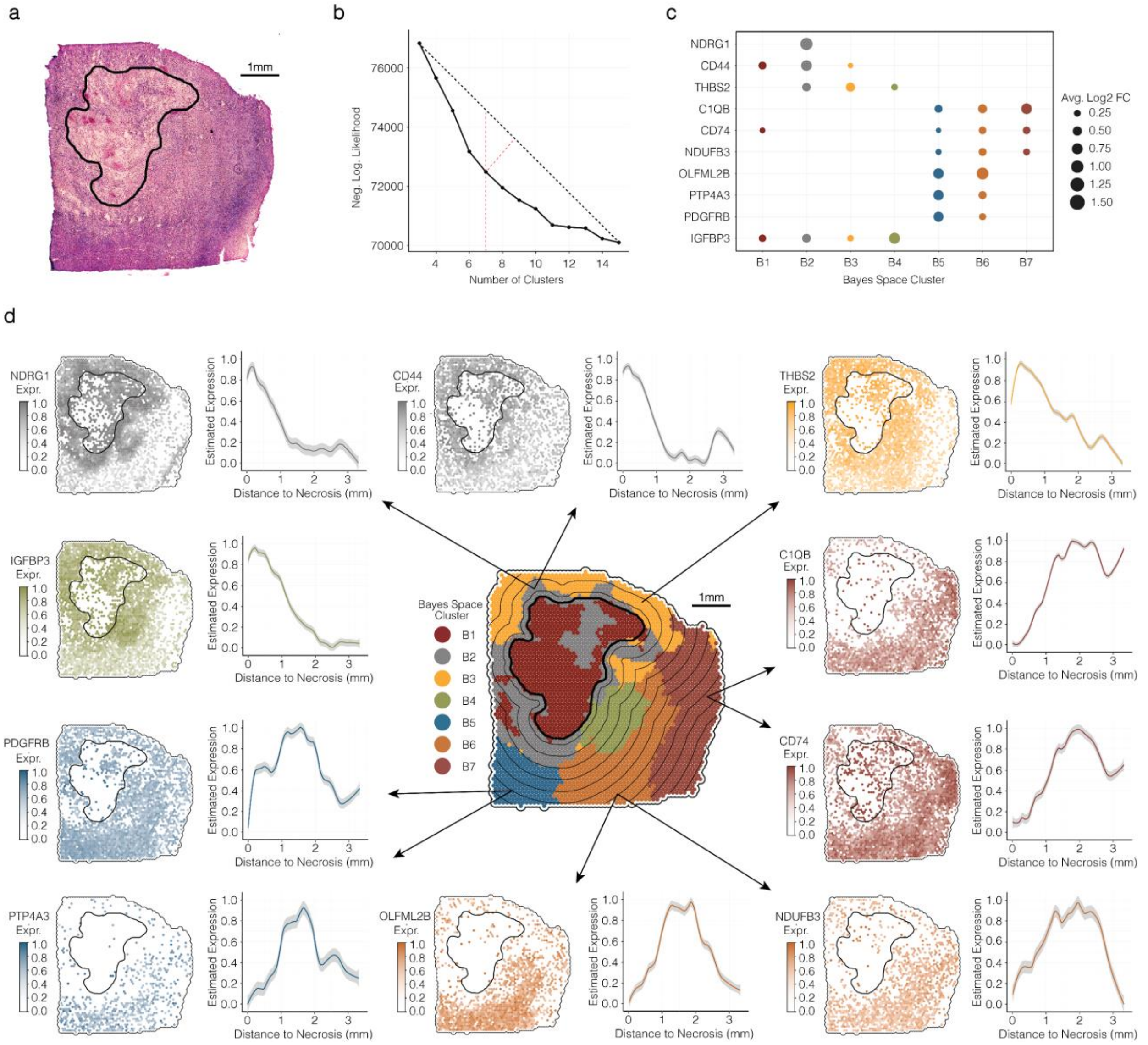

Supplementary Figure 4: Challenges in Clustering and Differential Gene Expression Analysis in a Spatial Transcriptomic Sample (UKF313T, Glioblastoma). This figure highlights complexities in clustering and differential gene expression analysis arising from a challenging gene expression architecture. a) Displays the Hematoxylin and Eosin (HE) stained tissue image, emphasizing the annotated necrotic area using SPATA2's spatial annotation concept. b) Depicts the negative logarithmic likelihood of different cluster numbers, determined computationally due to the absence of a clear elbow point. Seven clusters were selected as the optimal number. c) Presents a dot plot of representative marker genes for the seven clusters, revealing multiple genes highly upregulated across several clusters, leading to ambiguous cluster boundaries. d) Visualizes clustering results by BayesSpace (q=7) and indicates the assumed direction of gene expression gradients with circular expression estimates at specific distances. Further surface plots show the spatial gene expression of marker genes for each cluster. The color spectrum used for each corresponds to the cluster with the highest average log2 fold-change in differential expression analysis (DEA). Notably, high gene expression often extends beyond a single cluster highlighting the continuous and gradient like gene expression in pathological samples. Line plots illustrate gene expression relative to distance from the outlined necrotic center.

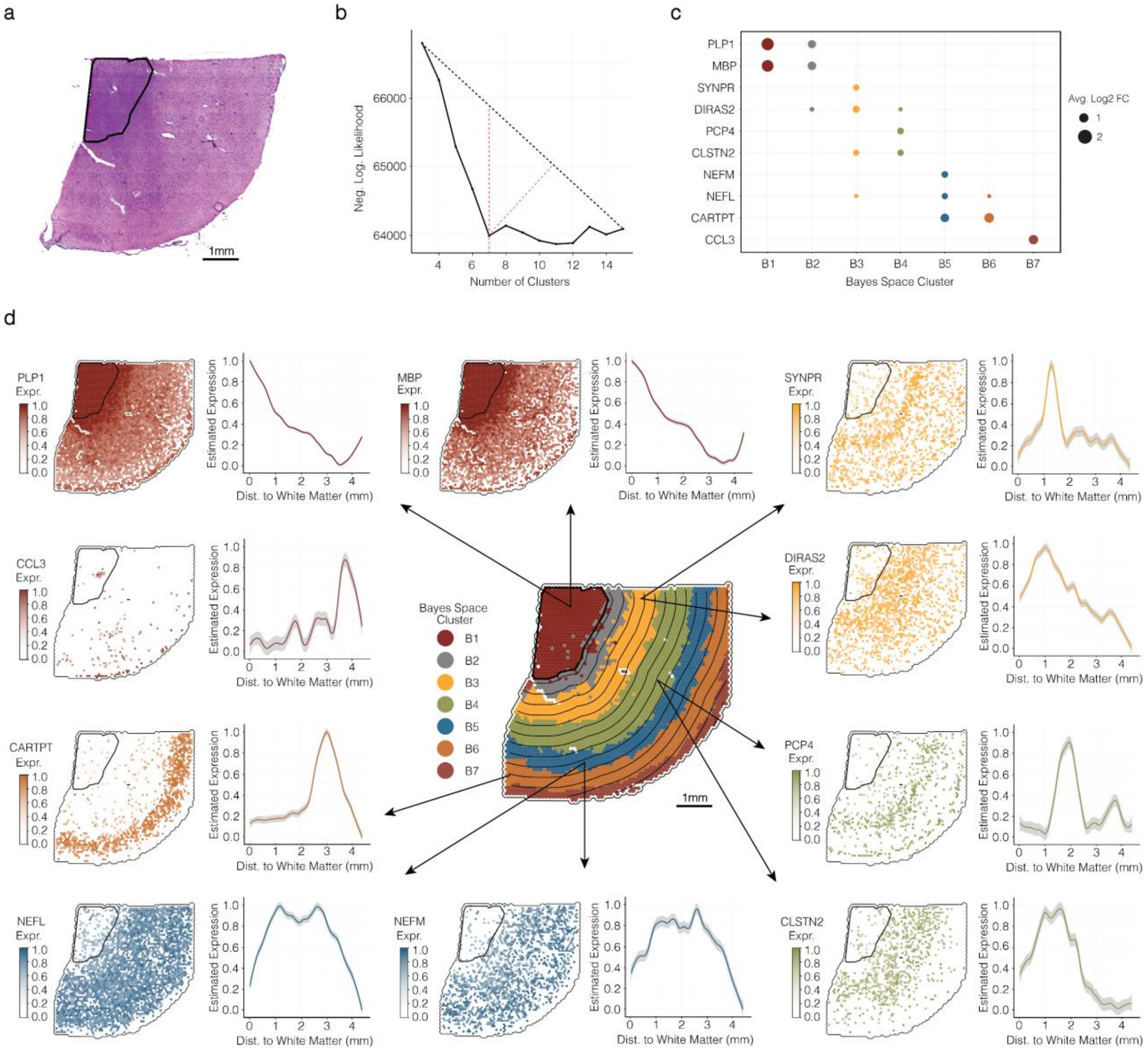

Supplementary Figure 5: Navigating Clustering and Differential Gene Expression Analysis in a Spatial Transcriptomic Sample (UKF265C, Human Neocortex). This figure elucidates the challenges and subtleties encountered in clustering and differential gene expression analysis, even when the spatial gene expression architecture is amenable to clustering techniques. a) Features the Hematoxylin and Eosin (HE) stained tissue image, highlighting the white matter region based on the cluster identified by BayesSpace using SPATA2's spatial annotation system. The area was used as reference in the plots displaying the gene expression gradient (d). b) Presents a plot displaying the negative logarithmic likelihood for varying cluster numbers, revealing a prominent elbow that suggests an optimal cluster count of seven. c) Displays a dot plot showcasing representative marker genes for each of the seven clusters. d) Visualizes clustering results by BayesSpace (q=7) and spatial gene expression of select marker genes. Gene colors correspond to the cluster with the highest average log2 fold-change in differential expression analysis (DEA). Notably, genes like SYNPR, PCP4, CARTPT, CCL3 exhibit a layered expression pattern with distinct peaks, while others such as PLP1, MBP, NEFM, and NEFL show more gradual variations across layers. Adjacent gradient plots illustrate gene expression in relation to the distance from the white matter zone borders, capturing the spatial dynamics of gene expression in the human neocortex.

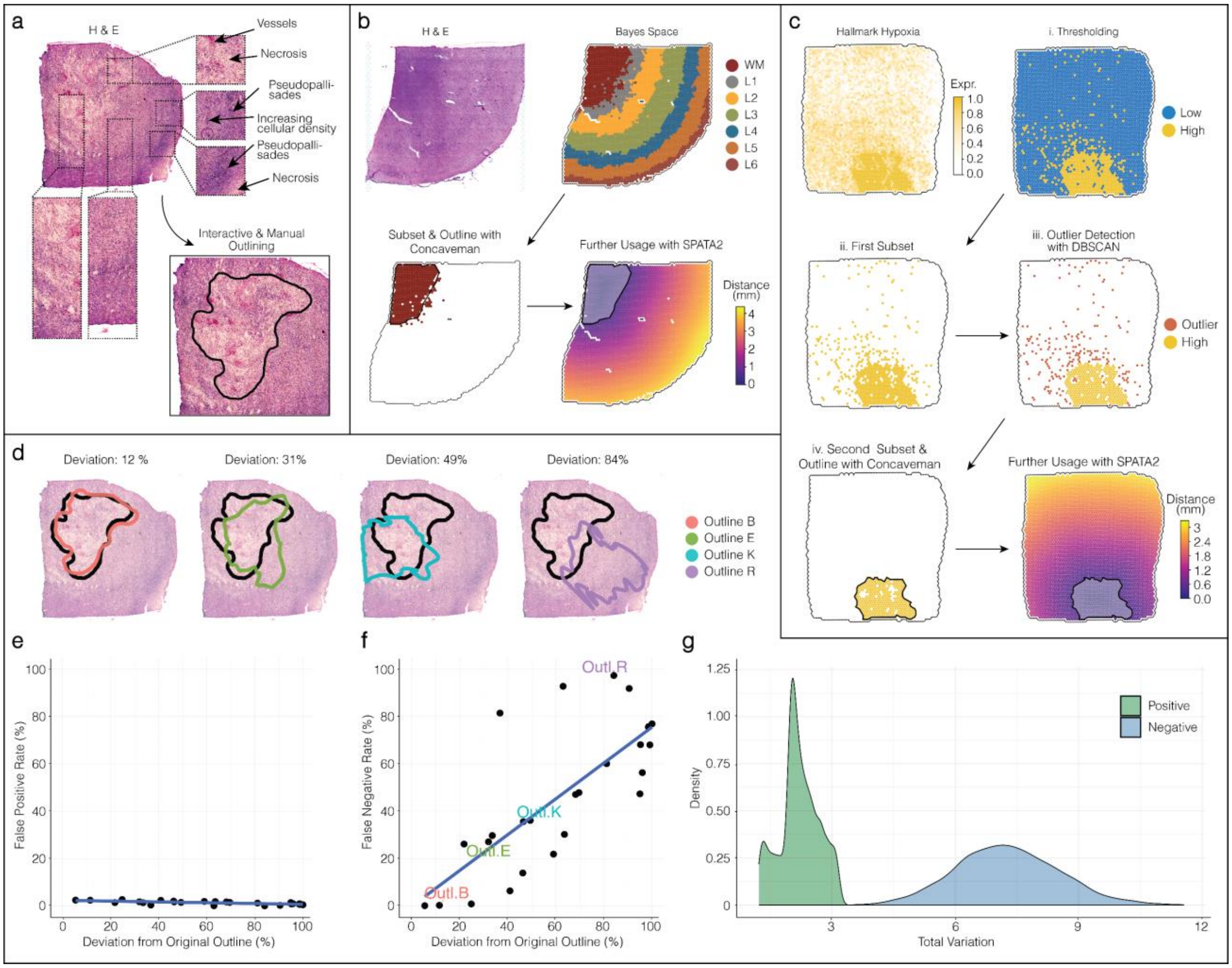

Supplementary Figure 6: Spatial Annotations and Investigation of Susceptibility to Human Bias. a) Demonstrates the concept of image annotations, where spatial outlines are manually generated through visual inspection of histomorphological features. b) Illustrates group-based spatial annotations, where outlines are created based on the positioning of data points within the same group. c) Presents numeric spatial annotations, where the expression of a selected feature forms the basis for spatial outline creation. d) Provides representative examples of outlines that progressively diverge from the original necrotic center outline. These examples were chosen from a collection of divergent outlines studied to investigate human bias. e) & f) Depict false positive and false negative rates in spatial annotation screening results with divergent outlines. Each point represents a run, positioned based on the degree of deviation from the original outline and corresponding test performance measures. The false positive rate remains nearly 0, while the false negative rate increases linearly with deviation from the original outline. Simulations with outlines deviating below 15% from the original outline exhibit no false negatives, indicating robustness within this range of deviation. g) Displays the distribution of total variation scores from the 4800 simulations used as ground truth for the screening runs in figures e and f.

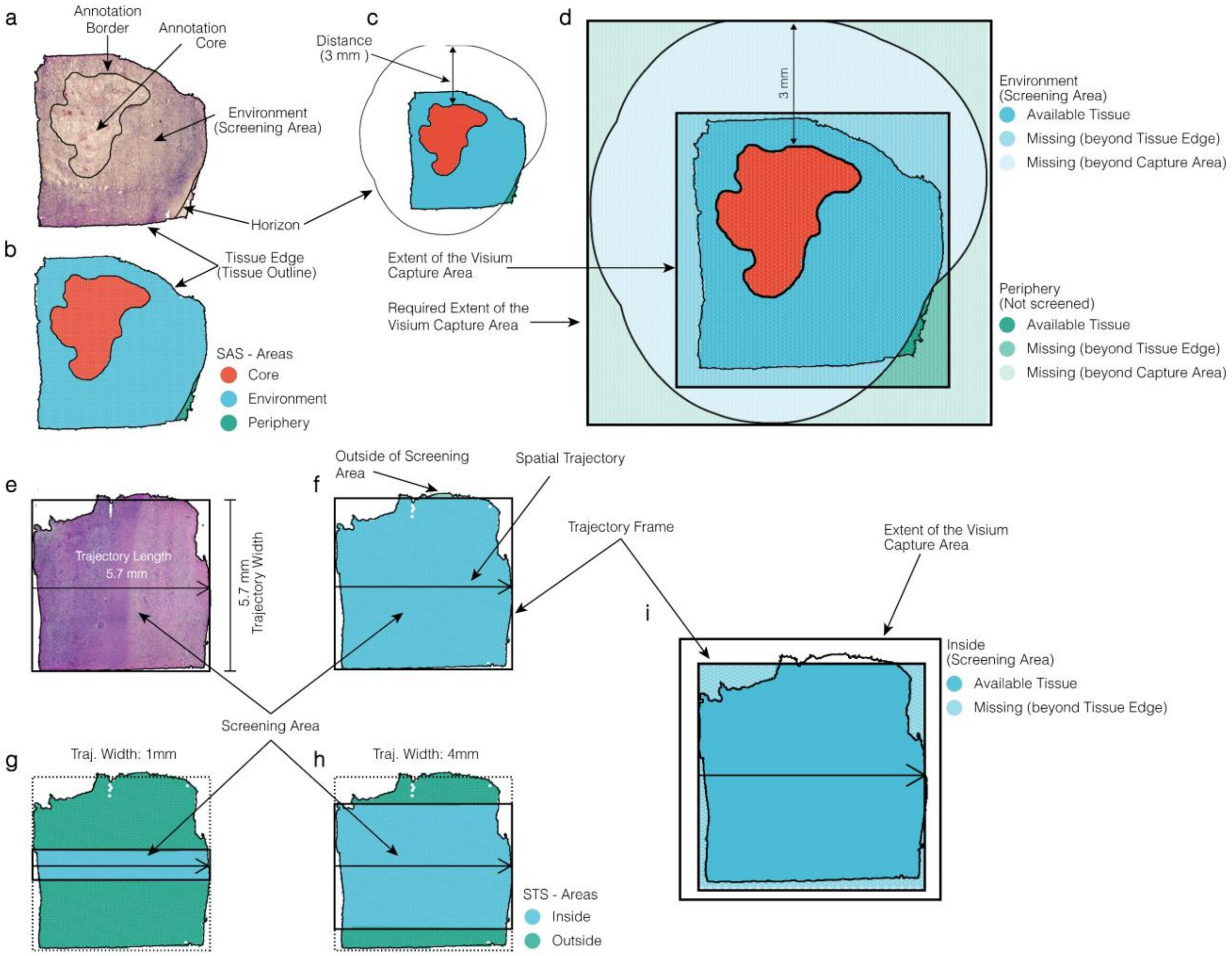

Supplementary Figure 7: Visual Glossary and Illustration of the Data Set Completeness in Spatial Gradient Screening. This figure serves as an illustrative guide to the terminology used in spatial gradient screening and the spatial reference objects used. Further, it demonstrates how data set completeness can vary based on the algorithm's input parameters leading to different correction factors for the screening resolution. (Note that a-d) refer to a screening set up that only references the large necrotic center and a distance up to 3mm. The set up used for Main Figure 4 referenced multiple necrotic areas.) a-b) These panels use labels and arrows to clarify the terms related to spatial annotations and spatial annotation screening, providing a visual reference for the vocabulary used in this context. c-d) These sections highlight the potential incompleteness of the data set when screening is conducted within a radius of 3mm. It is important to note that the core area is not included in the screening process, and thus its presence does not contribute to data completeness or incompleteness. e-h) In these panels, labels and arrows are used to define the terminology specific to spatial trajectories. Additionally, they visually depict the screening areas resulting from different inputs for trajectory width, offering a clear understanding of how varying parameters can influence the screening scope. i) This panel provides an illustration of the data set incompleteness specifically for the spatial trajectory used in our study, showcasing the limitations and gaps in data coverage that can arise in practical applications of the screening methodology.

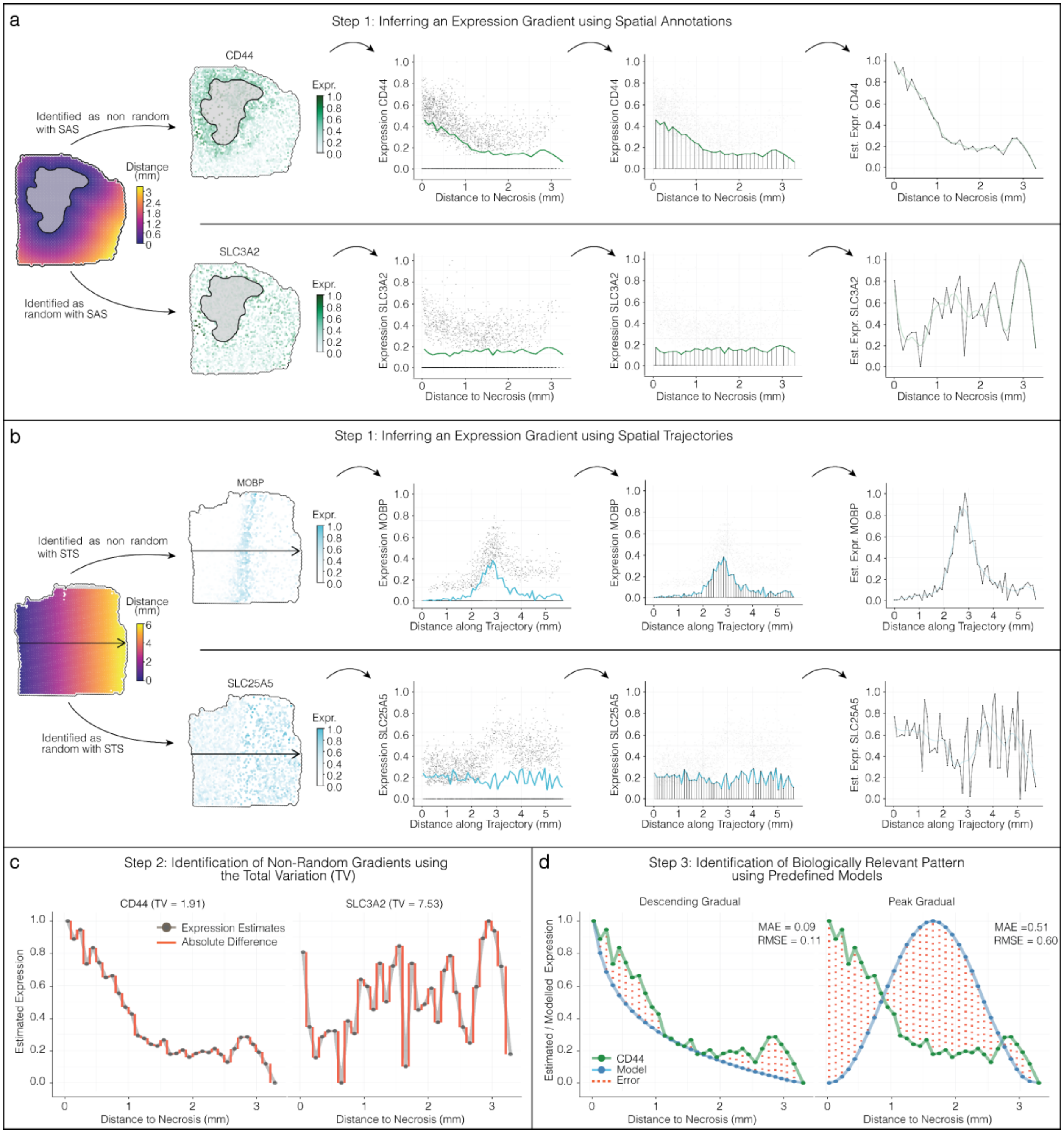

Supplementary Figure 8: Visual Representation of the Three Steps in Spatial Gradient Screening. a-b) Illustration of the first step within the context of spatial annotations (a) and spatial trajectories (b), using two distinct genes as examples—one identified as exhibiting a non-random pattern and another as a random pattern. Descriptions of each substep can be found in the methodology section. c) This panel delves into the second step of the spatial gradient screening algorithm, showcasing the application of the total variation score. This score quantifies the degree of randomness within a gradient, as demonstrated using examples of both non-random and random gradients from figure a). d) This panel illustrates the third step, encompassing model fitting, which employs the mean absolute error (or root mean squared error if the error is squared) as the evaluation metric. It contrasts a well-fitted model with one that does not closely match, emphasizing differences in fit. Errors, represented by dotted red lines, are more pronounced in the non-matching model (gradual peak) compared to a model closely aligned with the inferred gradient pattern from the gene CD44. The resultant evaluation is provided to the user, enabling the sorting of non-random genes based on their best-fitting model, thereby facilitating interpretation.

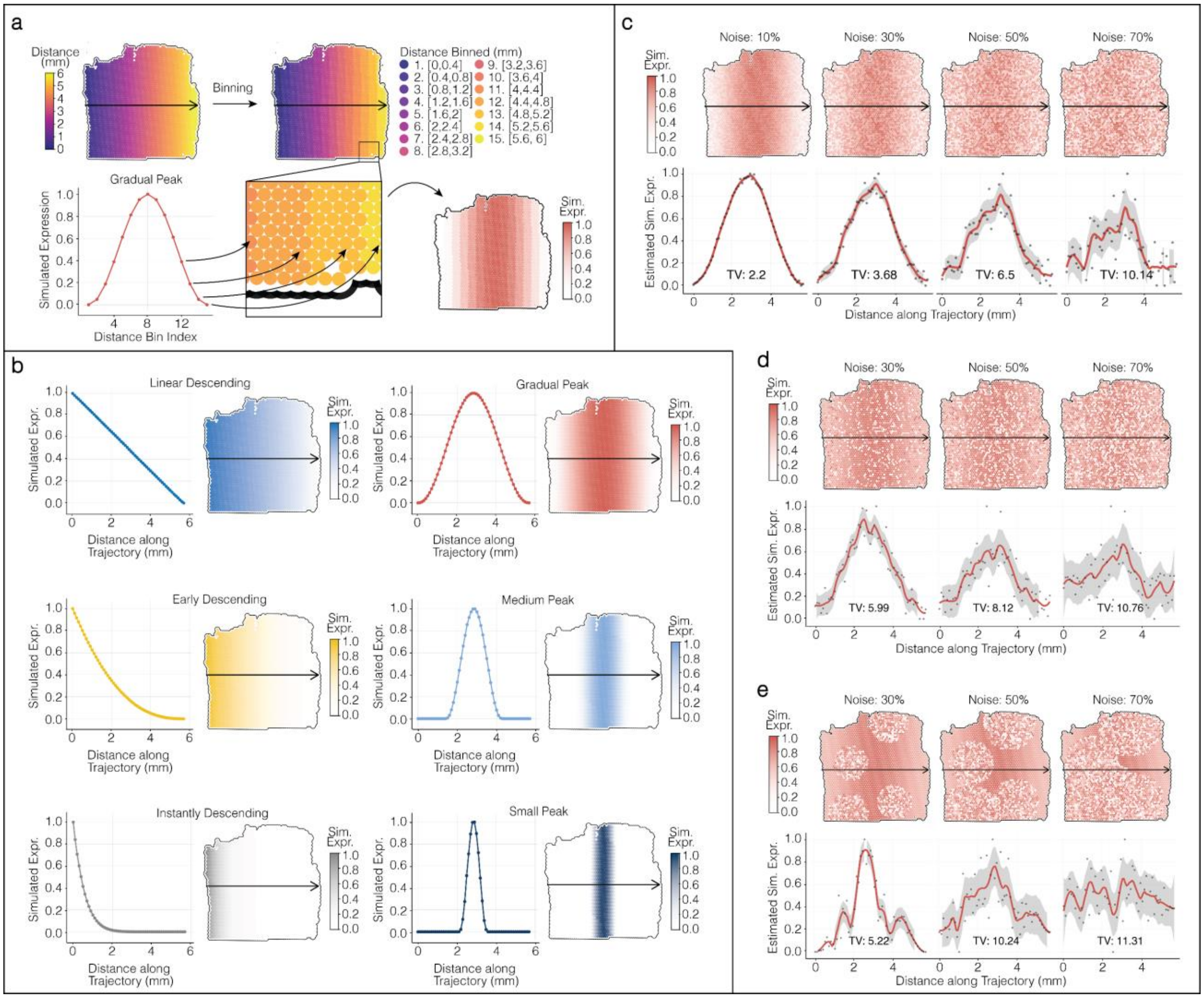

Supplementary Figure 9: Simulation of Gene Expression Along a Spatial Trajectory in Sample #UKF269T – Establishing Ground Truth for Analyzing Total Variation, Susceptibility to Human Bias, False Negatives, and False Positives. a) This panel offers a visual representation of the simulation concept. Refer to the methodology section for a comprehensive explanation. Note that the binning is presented at a binwidth of 400um for visualization purposes, as distinct groups at 100um intervals are not discernible to the human eye at this resolution. b) Emphasizes the selected underlying pattern of the simulations at 0% noise, using a binwidth of 100um. This is illustrated both as inferred gradients and through surface plots, providing a clear view of the pattern without interference from noise. c-e) Showcase the simulated expression for the 'gradual peak' pattern under varying percentages of noise, illustrating how expression changes under different noise conditions and its impact on the total variation score. Surface plots correspond to the line plots below. Points on the line plots represent expression estimates, with the line representing the smoothed pattern. Figure c) is dedicated to the 'equally distributed' noise type, demonstrating its effect on the expression pattern. Figure d) focuses on the 'equally punctuated' noise type, illustrating the influence of this specific noise distribution on expression. Figure e) exemplifies the 'focally punctuated' noise type, providing insights into how localized noise influences the simulated expression.

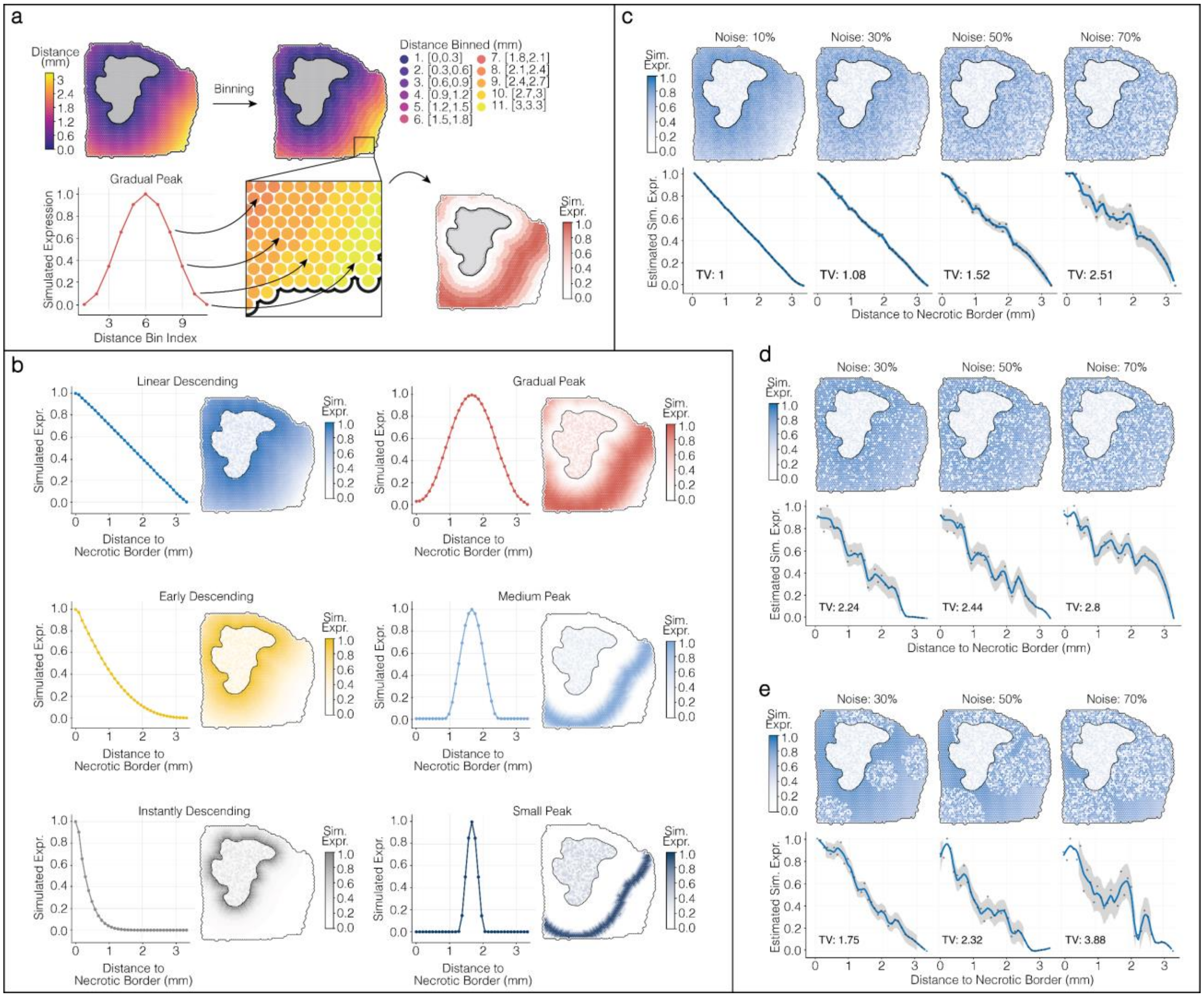

Supplementary Figure 10: Simulation of Gene Expression According to a Spatial Annotation in Sample #UKF313T – Establishing Ground Truth for Analyzing Total Variation, Susceptibility to Human Bias, False Negatives, and False Positives. a) This panel offers a visual representation of the simulation concept. Refer to the methodology section for a comprehensive explanation. Note that the binning is presented at a binwidth of 300um for visualization purposes, as distinct groups at 100um intervals are not discernible to the human eye at this resolution. b) Emphasizes the selected underlying pattern of the simulations at 0% noise, using a binwidth of 100um. This is illustrated both as inferred gradients and through surface plots, providing a clear view of the pattern without interference from noise. c-e) Showcase the simulated expression for the 'gradual peak' pattern under varying percentages of noise, illustrating how expression changes under different noise conditions and its impact on the total variation score. Surface plots correspond to the line plots below. Points on the line plots represent expression estimates, with the line representing the smoothed pattern. Figure c) is dedicated to the 'equally distributed' noise type, demonstrating its effect on the expression pattern. Figure d) focuses on the 'equally punctuated' noise type, illustrating the influence of this specific noise distribution on expression. Figure e) exemplifies the 'focally punctuated' noise type, providing insights into how localized noise influences the simulated expression.

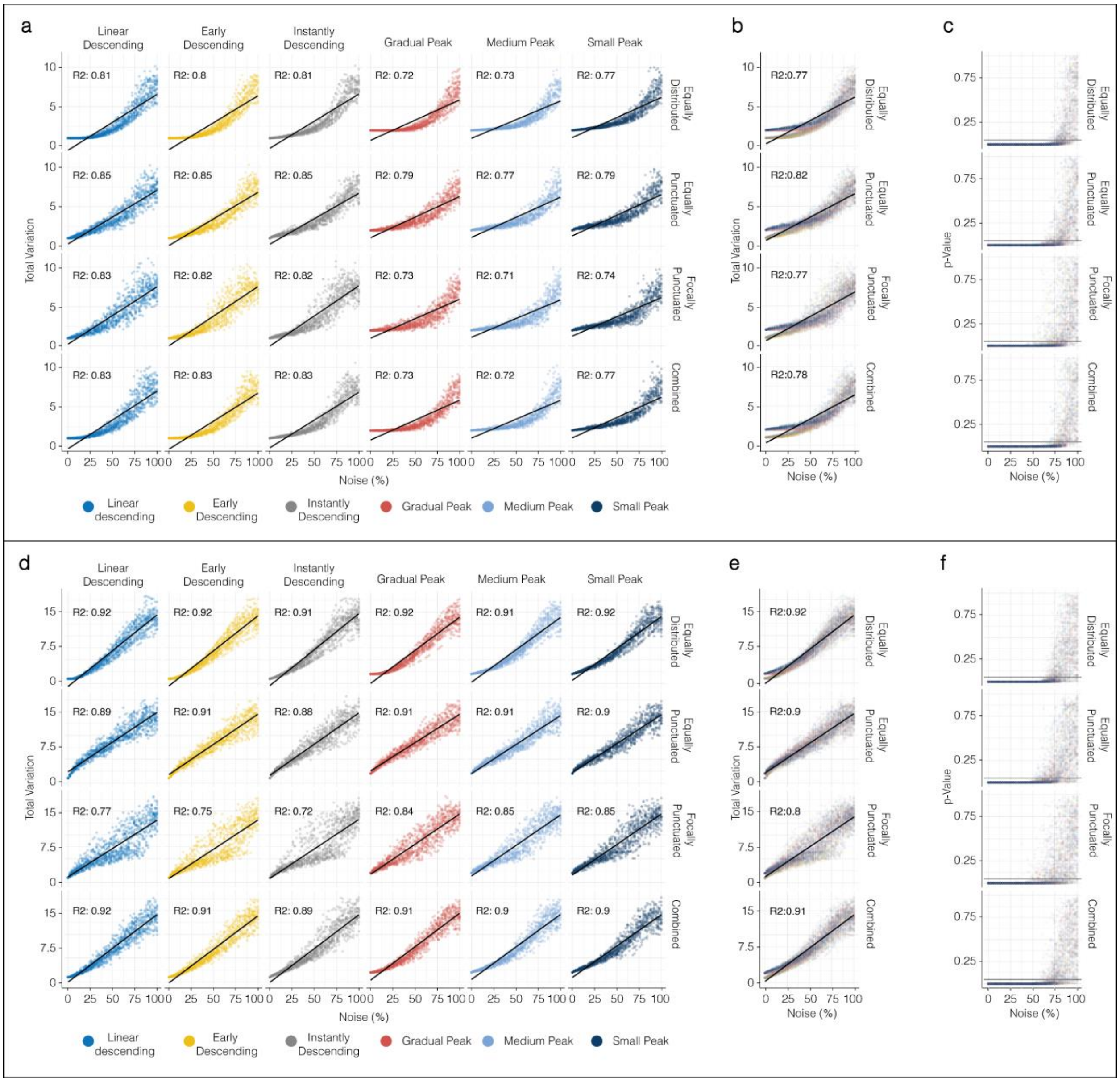

Supplementary Figure 11: Exploring Total Variation as an Indicator of Randomness in Inferred Gradients. Panels a-c) present the outcomes of simulations related to the spatial annotation of the necrotic center in sample #UKF313T. Panel d-f) focuses on the results from simulations based on the spatial trajectory in sample #UKF269T. Figures a and d), respectively for SAS and STS, illustrate the correlation between the percentage of noise introduced into a gradient and the corresponding total variation score, with sub-panels detailing the outcomes across different underlying patterns and noise types. Figures b and e), respectively for SAS and STS, consolidate these sub-panel results into one single comprehensive plot for each underlying type of noise. Figures c and f), respectively for SAS and STS, depict the p-values obtained in each simulation using the total variation metric, showcasing how these values correspond to the percentage of noise present in the gradient.

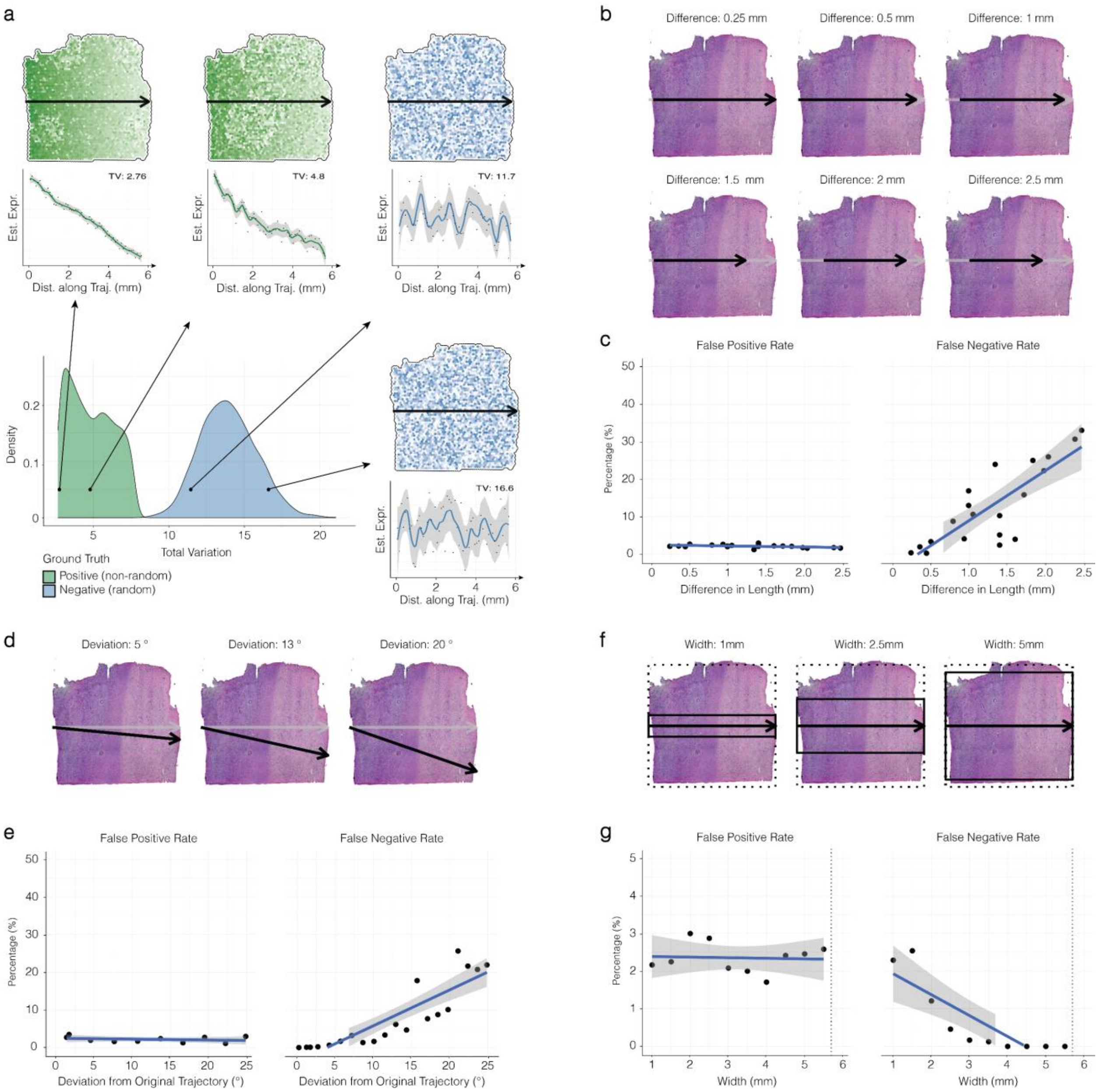

Supplementary Figure 12: Investigating Susceptibility to Human Bias in Spatial Trajectory Screening. a) Depicts the distribution of total variation scores for positive and negative cases within the ground truth used for investigations, with surface and gradient plots illustrating representative examples from both simulation groups. b-c) Present the results of sensitivity investigations regarding variations in start and endpoint. b) Features representative examples, while c) illustrates the relationship between false positives and false negatives in relation to differences in trajectory length resulting from shifts in the start and endpoint. d-e) Showcase the results of investigations into deviations from the original trajectory by degree. d) Highlights representative examples, while e) illustrates the correlation between false positives and false negatives and the degree of deviation from the original trajectory. f-g) Demonstrate the relationship between false positives and false negatives and changes in the screening area resulting from different width inputs. f) Features representative examples related to the default screening area width (matching the trajectory length). g) Highlights the relationship between false positives and false negatives and deviations from the original trajectory screening, measured by the width parameter input. Note the y-axis scale changes in comparison to the other figures.

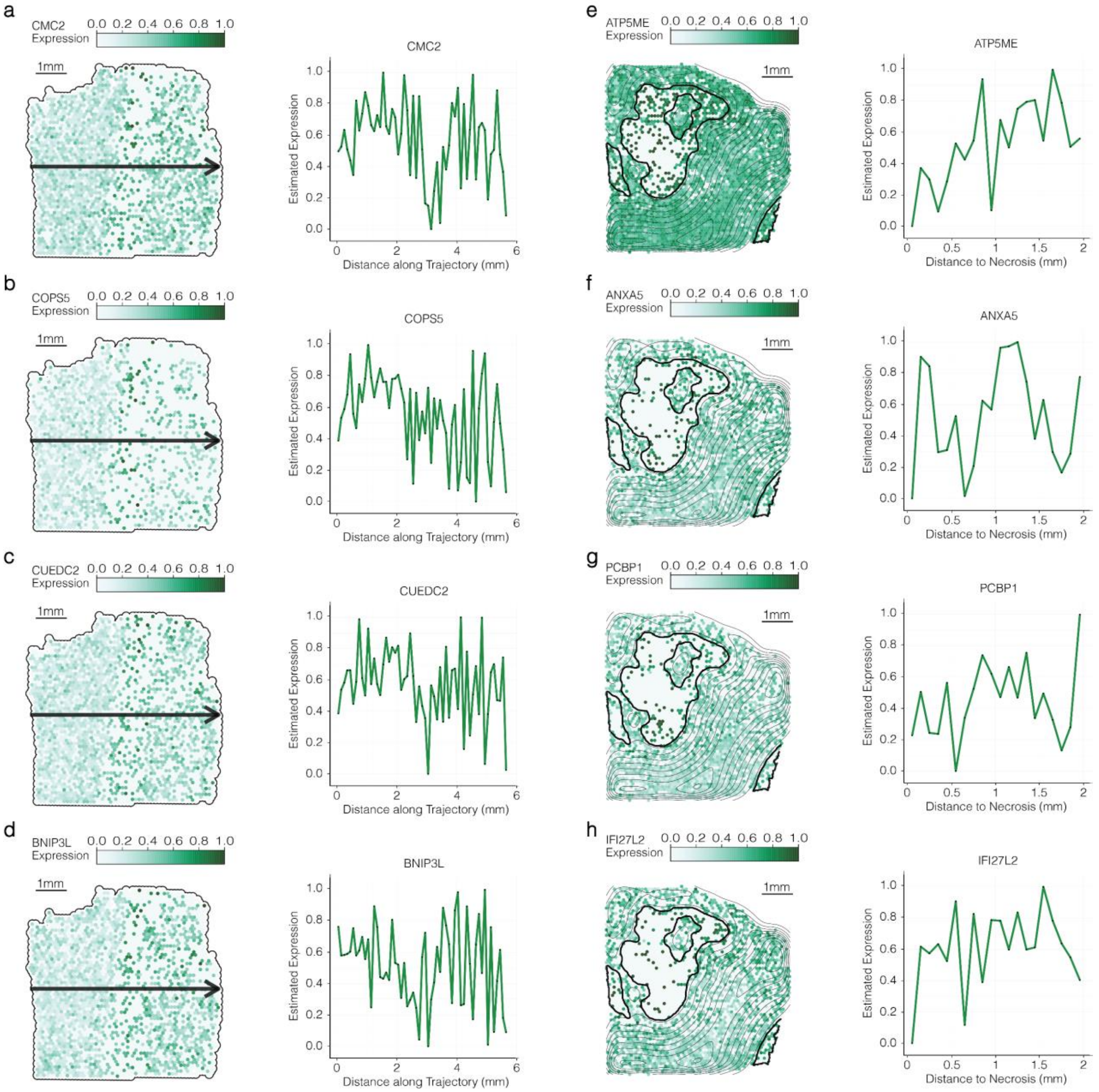

Supplementary Figure 13: Genes Identified as Statistically Spatially Significant by SPARKX, Yet Lacking Discernible Patterns Under Certain Assumptions. a-d) Surface plots with accompanying gradient plots of genes identified as statistically significant by SPARKX (adj. p-value < 0.0001) but exhibiting no specific association with the tumor-transition-cortex architecture from sample UKF269T. e-h) Surface plots with associated gradient plots of genes identified as statistically significant by SPARKX (adj. p-value < 0.0001) but displaying no evident pattern relative to distance from the three necrotic annotations from sample UKF313T, suggesting their independence from the presence and dynamics surrounding necrosis.

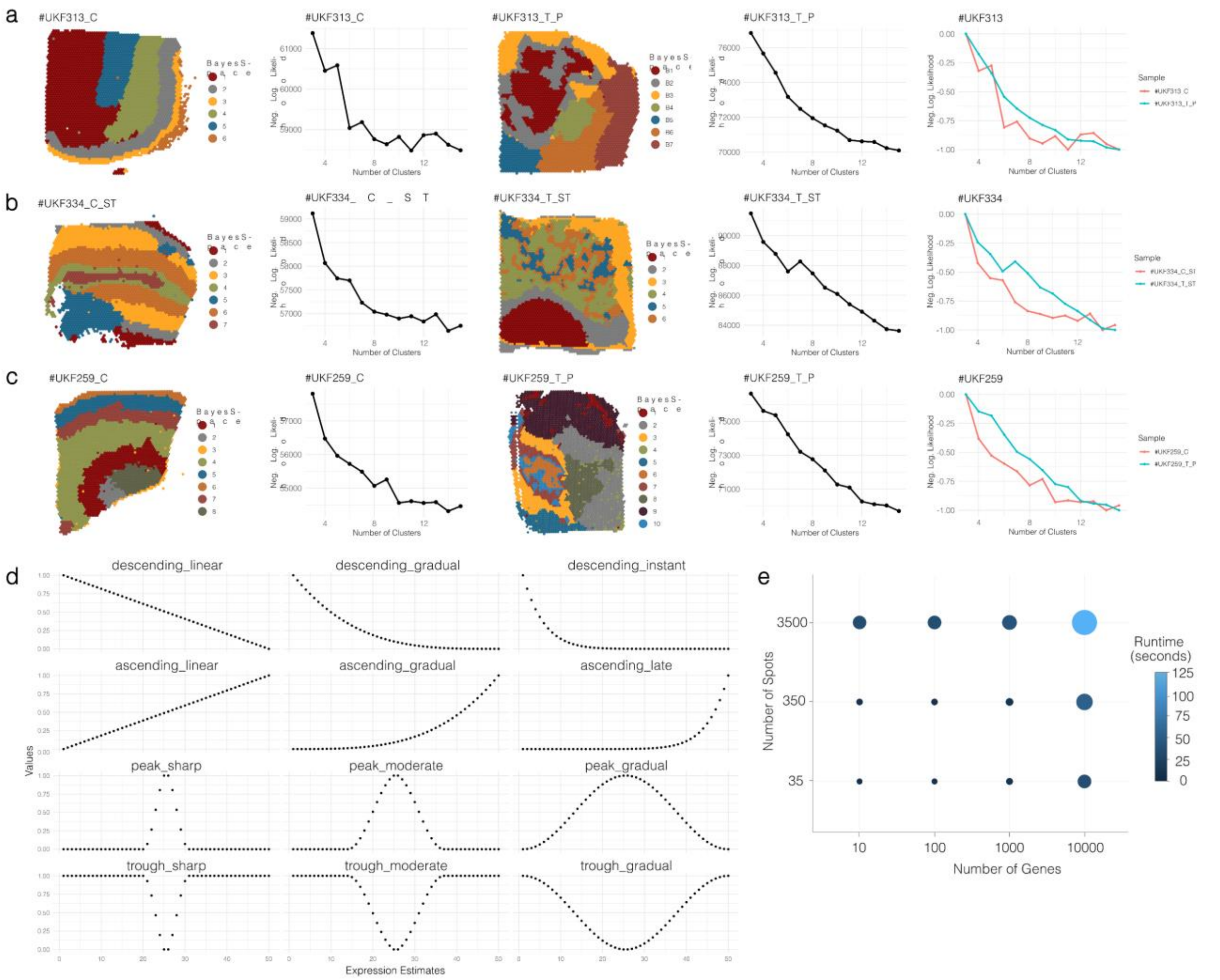

Supplementary Figure 14: Comparative BayesSpace Clustering Analysis in Cortical Sections of Healthy and Glioblastoma Tissue from the Same Patients: a-c) Comparing BayesSpace clustering in cortical sections of healthy versus glioblastoma tissue of the same individuals. Shown are 10X Visium sections from three exemplary patients (one per row). For each patient, plots depict the following (left to right): Surface plot of BayesSpace clusters in healthy tissue; q-plot of healthy tissue (that is, the number of BayesSpace clusters plotted against the negative log-likelihood of Bayesspace q.logliks); surface plot of of BayesSpace clusters in diseased tissue; q-plot of diseased tissue; overlay of q-plots from healthy and diseased tissue of the same patient. d) Exemplary models that can be used for screening in SPATA2. Models represent simple numeric vectors that are customizable. e) Benchmark of SPATA2::spatialAnnotationScreening() adjusting the screening distance to include differing number of spots and including different number of genes (Runtime corresponds to the median time required from 15 iterations).
